# Validation of the Composite Autonomic Symptom Score 31 in the German language

**DOI:** 10.1007/s10072-021-05764-4

**Published:** 2021-11-25

**Authors:** Max-Josef Hilz, Ruihao Wang, Wolfgang Singer

**Affiliations:** 1 Present Address: Department of Neurology, University of Erlangen-Nuremberg, Schlossplatz 4, 91054 Erlangen, Germany; 2 Icahn School of Medicine at Mount Sinai, New York, NY USA; 3 Present Address: Department of Neurology, Mayo Clinic, Rochester, MN USA

**Keywords:** COMPASS 31, Autonomic symptoms, German validation, Self-questionnaire

## Abstract

**Background:**

The Composite Autonomic Symptom Score 31 (COMPASS 31) is a validated, 31-item self-assessment questionnaire assessing autonomic symptoms in six domains, orthostatic intolerance, vasomotor, secretomotor, gastrointestinal, bladder, and pupillomotor function. So far, there is no validated German COMPASS 31 version. This study aimed at developing and validating a German COMPASS 31.

**Methods:**

Two autonomic experts with command of German and English independently translated the English COMPASS 31 into German. One agreed-upon German version was translated back into English to assure conformity with the original version. Twenty patients with possible autonomic symptoms and 20 age- and gender-matched healthy persons completed the English and German COMPASS 31 in a randomized order with a 4-week interval. To evaluate reliability of the German COMPASS 31, total scores and sub-scores of the domains assessed with the German version were correlated with corresponding scores of the English version using Pearson’s or Spearman’s test. The Cronbach alpha-coefficient evaluated the internal consistency of the questions. Total- and sub-scores of both COMPASS 31 versions were compared between patients and controls by analysis of variance with post-hoc analysis (significance: *p* < 0.05).

**Results:**

Total scores and sub-scores of the German and English COMPASS 31 correlated significantly (*p* < 0.001) and closely (correlation coefficients: 0.757–0.934). Cronbach alpha-coefficients were above 0.7 in all domains except for the secretomotor domain. In the German and English COMPASS 31, total scores were significantly higher in patients than controls.

**Conclusions:**

The German COMPASS 31 is reliable, internally consistent, and valid to detect and quantify autonomic symptoms in patients with neurological disorders.

**Supplementary Information:**

The online version contains supplementary material available at 10.1007/s10072-021-05764-4.

## Introduction

Autonomic manifestations such as orthostatic intolerance, urogenital symptoms, and gastrointestinal complaints are common among patients with various neurological disorders, e.g., movement disorders [1], neuro-immunological diseases [2], cerebrovascular diseases [3], and neuro-degenerative diseases [4]. Surprisingly, these autonomic symptoms are quite often overlooked by physicians in the routine clinical practice. The Composite Autonomic Symptom Score 31 (COMPASS 31) is a validated, 31-item self-assessment questionnaire, aiming to assess the global autonomic symptoms [5]. It was originally developed by Singer, Sletten and colleagues from the Mayo Clinic autonomic group [5], based on the more time-consuming 169-item Autonomic Symptom Profile (ASP) and the 84-question scoring instrument COMPASS [6]. In COMPASS 31 self-assessment questionnaire, the 31 items evaluate global autonomic symptoms in 6 domains, i.e., orthostatic intolerance, vasomotor, secretomotor, gastrointestinal, bladder, and pupillomotor symptoms [6]. Compared with ASP and COMPASS, the COMPASS 31 scale is not only more user-friendly for the patients, its scoring algorithm is also much easier for physicians to perform [5].

Since its original publication in 2012, the COMPASS 31 has been applied in assessing autonomic symptoms in patients with neurological disorders as well as other related diseases [7, 8]. Recently, the original US English version of COMPASS 31 has been validated in the Italian language [9] and in the Croatian and Serbian languages [10].

So far, there is no validated German version of the COMPASS 31 questionnaire. Therefore, in this study, we aimed to develop a validated COMPASS 31 questionnaire in the German language.

## Methods and study participants

### Translation of the COMPASS 31 form the US English into German

Two neurologists with expertise in the autonomic nervous system who are both native German speakers with proficient command of the English language (WS and MJH) independently translated the original US English version of the COMPASS 31 questionnaire [5] into German. The two autonomic neurologists subsequently cooperated to create one German version. Then, this version was translated back into English to assure the conformity of the German version with the original US English version. In the end, WS and MJH produced the final German version of COMPASS 31 questionnaire (as supplemental resource uploaded).

### Study participants

At the Department of Neurology, University of Erlangen-Nuremberg, Erlangen, Germany, we enrolled 20 patients with neurological disorders that may be associated with autonomic symptoms and 20 age- and gender-matched healthy participants. Since we wanted to avoid testing patients who might have autonomic dysfunction in only one or two of the six COMPASS 31 domains, we enrolled patients with different diseases. However, we intended to assure that we avoided any influence on the patients’ responses to the 31-item questionnaire as well as any bias of the medical staff explaining the study objective and questionnaire to the participants of the validation study. Therefore, patients received the questionnaire prior to the subsequent detailed neurological work-up. All the study participants were bilingual, with German as native language and fluent in English.

The 40 study participants completed the COMPASS 31 twice, after an interval of four weeks ± one week, once in German and once in US English. The order of completing the questionnaire was randomized at the Department of Neurology, Mayo Clinic, Rochester, MN, USA, and an entry number that determined the order of completing the German or the English version was sent to us. We only enrolled patients with relatively stable clinical disease course, and with no planned major changes in the management during this 4-week period.

The study was approved by the Ethics Committee of the University of Erlangen-Nuremberg, Erlangen, Germany. All participants had signed the informed consent according to the Declaration of Helsinki.

### Data collection and Scoring Algorithm of COMPASS 31

The COMPASS 31 questionnaire consists of 31 items that cover 6 domains of autonomic symptoms, including 4 items on the orthostatic intolerance domain, 3 items on the vasomotor domain, 4 items on the secretomotor domain, 12 items on the gastrointestinal domain, 3 items on the bladder domain, and 5 items on the pupillomotor domain. Detailed information on the items of questions is described in the original publication of the US English Version of COMPASS 31 [5]. Each answer provided for an item is first assigned to a raw score; the sum of all raw scores assigned to the items covering one of the six domains is considered the sub raw score of this particular domain. Then, this sub raw score of each of the six domains is converted into the weighted sub-score by multiplying the raw score with a so-called weighting factor (which is derived from the relevance of each domain for assessing autonomic function) [5]. The sum of the six weighted sub-scores yield the total weighted score which ranges from 0 to 100, with 0 meaning no autonomic symptoms, and 100 reflecting the most severe autonomic symptoms [5]. We uploaded all collected data to Redcap, a secure online database system at Mayo Clinic, Rochester, MN, USA.

### Statistical analysis

Kolmogorov–Smirnov test was used to assess whether the data were normally distributed or not. We tested age-differences between patients with neurological disorders and healthy participants by Student’s *t*-test, and compared differences in the gender ratio between the two groups by the chi-squared test. To assess the reliability of the translated, German version of the COMPASS 31, we correlated the total weighted scores and the weighted sub-scores of the German COMPASS 31 version with the respective weighted scores in the original US English version, using Pearson’s correlation for normally distributed scores and Spearman’s rank correlation for non-normally distributed scores. For the German version and for the US English version of the COMPASS 31, we calculated Cronbach’s alpha coefficient for each of the six sets of questions covering one of the six COMPASS 31 domains to determine the internal consistency of each set of questions. Cronbach’s alpha coefficient values of 0.7 or higher are considered to indicate that there is an acceptably close relation among the questions within a given domain-specific set of questions, and thus sufficient reliability of the questions. To compare the weighted total and sub-scores of the US English version and German version of the COMPASS 31 between patients and healthy participants, we performed analysis of variance for repeated measurements (RANOVA), with “language” (“US English” or “German”) as a within-subject factor, and “group” (“patients with neurological disorders” or “healthy participants”) as between-subject factor. We applied the Mauchly’s Test of Sphericity to evaluate suitability of the ANOVA model and employed the Greenhouse Geisser correction in case of violation of the sphericity assumption. In case of significant RANOVAs, we performed post hoc single comparisons using non-paired Student’s *t*-tests for comparison between groups and paired Student’s *t*-tests for comparison between two languages if data were normally distributed. In case of not normally distributed data, we performed the Mann–Whitney *U*-test for independent samples to compare values of patients and healthy participants, and the Wilcoxon signed-rank test to assess whether there was any significant difference between weighted total scores and weighted sub-scores derived from the responses to the German version and the responses to the US English version of the self-questionnaire. We used a commercially available statistical program (SPSS, IBM SPSS Statistics 20) for data analysis. Statistical significance was assumed for *P*-values below 0.05.

## Results

### Demographic data of the study participants

Among the 20 patients participating in the study, eight patients had already known Parkinson’s disease (PD), four patients had diabetic autonomic neuropathy (DAN), one patient had Sjörgen syndrome, one patient had known multiple system atrophy (MSA), and one patient had Guillain-Barré syndrome. Five of the twenty patients had been referred because of orthostatic hypotension (OH) of unknown etiology. Age, gender, and interval of the two evaluations did not differ between the 20 patients with neurological disorders and the 20 healthy participants (Table 1).

**Table 1:** Demographic data of 20 patients with neurological disorders and 20 healthy participants

	Patients with neurological disorders (*n* = 20)	Healthy participants (*n* = 20)	*P*-value
Age (year)	52.1 ± 19.1	45.7 ± 14.5	0.234 ^a^
Gender (women/men)	11/9	10/10	1.000 ^b^
Interval between two evaluations (days)	32.1 ± 4.5	31.0 ± 4.9	0.464 ^a^

Age, gender distribution, and intervals between filling the German and English COMPASS 31 version in a randomized order did not differ between the patients and the healthy participants. Data are expressed as mean ± standard deviation; a = Student’s *t*-test, b = Chi-squared test

### Reliability of the German version of COMPASS 31

**Fig. 1:**
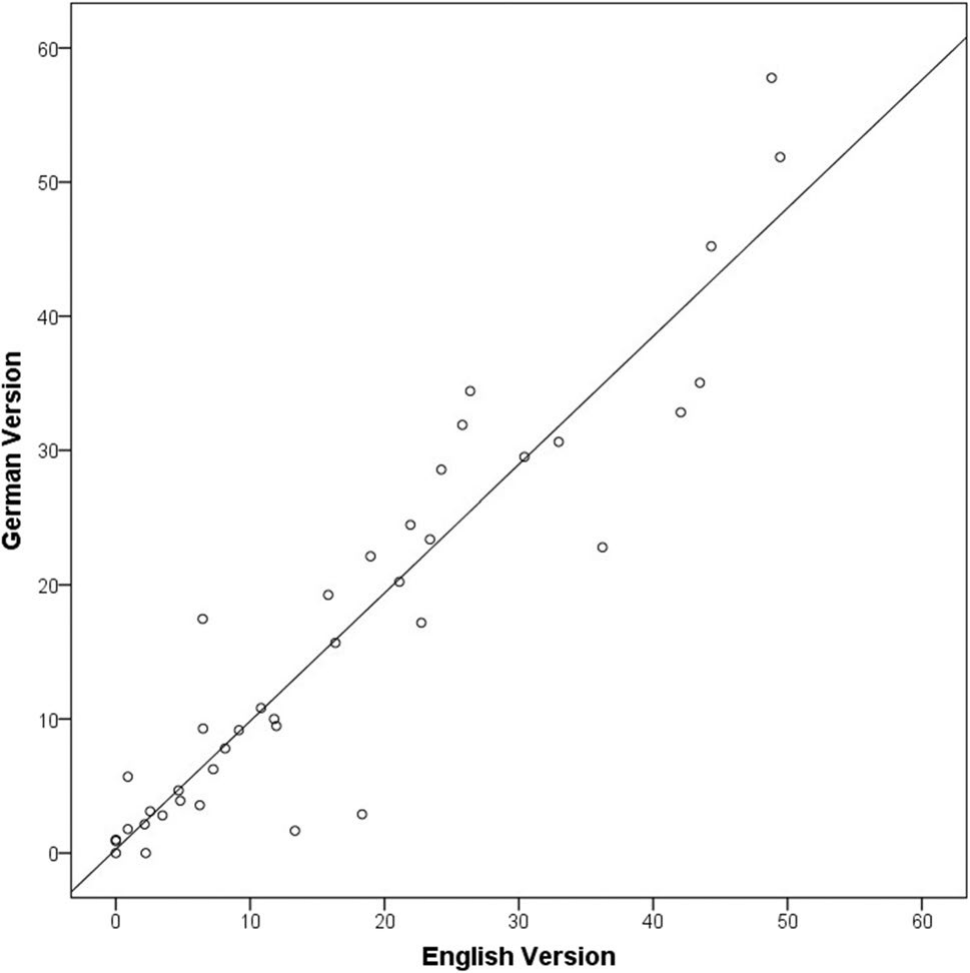
Close correlation between the total scores assessed with the German and the English versions of the COMPASS 31 questionnaire (*n* = 40). COMPASS 31, Composite Autonomic Symptom Score 31. Pearson’s correlation coefficient *r* = 0.934; *P* < 0.001

Among the total 40 study participants, the German version of COMPASS 31 significantly and positively correlated with the original US English version of COMPASS 31 for the total weighted score (coefficient = 0.934, *p* < 0.001), as well as in all weighted sub-scores: the orthostatic intolerance domain (coefficient = 0.919, *p* < 0.001), the vasomotor domain (coefficient = 0.900, *p* < 0.001), secretomotor domain (coefficient = 0.724, *p* < 0.001), gastrointestinal domain (coefficient = 0.896, *p* < 0.001), bladder domain (coefficient = 0.760, *p* < 0.001), and pupillomotor domain (coefficient = 0.757, *p* < 0.001; Fig. 1; Table 2).

**Table 2:** Correlation between total and sub-domain scores in the German and English COMPASS 31 versions and Cronbach alpha coefficients of both COMPASS 31 versions assessed in 40 study participants

Domain	Correlation between German and English COMPASS 31 scores		Cronbach alpha coefficients for all 31 questions and the six sets of sub-domain questions	
	Correlation coefficient	*P*-value	German version	US English version
Total scores	0.934 ^a^	< 0.001	0.881	0.865
Orthostatic intolerance	0.919 ^a^	< 0.001	0.907	0.895
Vasomotor	0.900 ^b^	< 0.001	0.901	0.920
Secretomotor	0.724 ^b^	< 0.001	0.676	0.536
Gastrointestinal	0.896 ^a^	< 0.001	0.775	0.791
Bladder	0.760 ^b^	< 0.001	0.850	0.875
Pupillomotor	0.757 ^a^	< 0.001	0.855	0.833

a = Pearson’s correlation, b = Spearman’s rank correlation

*COMPASS 31* Composite Autonomic Symptom Score 31

### Internal consistency of the German version of COMPASS 31

The values of Cronbach alpha coefficient of the German version of COMPASS 31 were 0.907 for the orthostatic intolerance domain, 0.901 for the vasomotor domain, 0.676 for the secretomotor domain, 0.775 for the gastrointestinal domain, 0.850 for the bladder domain, 0.855 for the pupillomotor domain, and 0.881 for the entire 31 questions. The values of Cronbach alpha coefficient of the original US English version of COMPASS 31 were 0.895 for the orthostatic intolerance domain, 0.920 for the vasomotor domain, 0.536 for the secretomotor domain, 0.791 for the gastrointestinal domain, 0.875 for the bladder domain, 0.833 for the pupillomotor domain, and 0.865 for the entire questionnaire (Table 2).

### Comparisons of the COMPASS 31 scores between patients and controls

In the German and the original US English versions, the weighted total scores of the COMPASS 31 were significantly higher in the patients with neurological disorders than in the healthy participants (Table 3). In the German as well as the US English versions of the COMPASS 31, the weighted sub-scores in the orthostatic intolerance domain and the pupillomotor domain were slightly but not significantly higher in the patients than in the healthy participants, with *p*-values for both domains in the German and English version ranging from 0.064 to 0.081 (Table 3). The other COMPASS 31 sub-scores did not differ significantly between patients and healthy participants in either language version of the COMPASS 31 (Table 3).

**Table 3:** Comparison of total scores and sub-scores assessed with the original English and the German COMPASS 31 versions in 20 patients with neurological disorders and 20 healthy participants

Domain	Version	Patients with neurological disorders (n = 20)	Healthy participants (*n* = 20)	*P*-value Patients vs. controls
Total scores	English Version	**22.0 ± 17.9**	**11.8 ± 8.2**	**0.025**
	German Version	**21.6 ± 18.1**	**11.2 ± 9.1**	**0.028**
		*P* = 0.732	*P* = 0.684	
Orthostatic intolerance	English Version	**11.8 ± 12.9**	**5.8 ± 6.8**	**0.076**
	German Version	**11.0 ± 12.4**	**5.1 ± 7.5**	**0.079**
		*P* = 0.297	*P* = 0.565	
Vasomotor	English Version	0.3 ± 0.8	0.3 ± 0.8	0.799
	German Version	0.2 ± .7	0.3 ± 0.7	0.620
		*P* = 0.317	*P* = 0.317	
Secretomotor	English Version	2.5 ± 3.2	1.3 ± 1.8	0.512
	German Version	2.6 ± 2.6	1.4 ± 2.1	0.495
		*P* = 0.847	*P* = 0.772	
Gastrointestinal	English Version	4.8 ± 4.1	3.3 ± 2.6	0.171
	German Version	5.2 ± 4.1	3.8 ± 2.9	0.212
		*P* = 0.180	*P* = 0.203	
Bladder	English Version	1.2 ± 2.4	0.1 ± 0.5	**0.060**
	German Version	1.1 ± 1.7	0.3 ± 0.9	**0.072**
		*P* = 0.649	*P* = 0.330	
Pupillomotor	English Version	**1.5 ± 1.1**	**0.9 ± 0.8**	**0.081**
	German Version	**1.5 ± 1.3**	**0.9 ± 0.9**	**0.064**
		*P* = 0.931	*P* = 0.549	

Data are expressed as mean ± standard deviation. *P*-values of the comparison between the English and German version scores are shown below the scores. *P*-values of the comparison between the scores of patients and of controls were listed in the right column. Values of significant differences or with a tendency of significance were expressed as bold.

*COMPASS 31* Composite Autonomic Symptom Score 31

In both the patients group and the healthy controls group, total scores as well as sub-scores of COMPASS 31 did not differ between the original US English and the German versions (Table 3).

## Discussion

Our study results show three major findings that confirm that the German version of the COMPASS 31 provides valid and reliable data that do not differ from results obtained with the original US English version of the questionnaire. First, the total- and sub-scores obtained in our patient and control group with the German COMPASS 31 version show a close and high correlation with the scores assessed with the original US English COMPASS 31 (Table 2). Thus, we can conclude that the reliability of the German COMPASS 31 version does not differ from the high reliability demonstrated in various studies for the US English COMPASS 31 (Sletten, Suarez et al. 2012; Treister, O'Neil et al. 2015).

Second, the German version of the COMPASS 31 showed a high internal consistency with Cronbach’s alpha values that were similar to the Cronbach’s alpha values of the original COMPASS 31. The Cronbach’s alpha coefficients for the orthostatic intolerance, secretomotor, pupillomotor domain, and for the entire questionnaire were even slightly higher than the corresponding coefficients of the US English version (Table 2). Thus, the internal consistency of each set of German questions evaluating the six autonomic domains is high and shows the same reliability of the questions as does the US English COMPASS 31.

Third, the German COMPASS 31 version identifies and quantifies autonomic dysfunction of various organs and functional systems with a high sensitivity, similar to the US English version (Sletten, Suarez et al. 2012; Treister, O’Neil et al. 2015). Both the US English and the German COMPASS 31 yielded significantly higher total COMPASS 31 scores in the 20 patients with diverse neurological disorders than in 20 healthy participants, and the scores of the six domains were again similar in the German and the US English version (Table 3). The finding that there was only a trend towards higher sub-scores in the patients than the controls for the orthostatic, bladder, and pupillomotor domains, with *p*-values ranging between 0.064 and 0.081 (Table 3), and no significant difference between sub-scores of patients and controls in the vasomotor, secretomotor, and gastrointestinal domains (Table 3) is most likely due to the heterogeneity of neurological disorders evaluated in our study and the resulting small samples of disease entities. Our patient selection was associated with rather small numbers of patients with a specific disease; e.g., there was only one patient with Sjörgen syndrome, one patient with multiple system atrophy, and one patient with Guillain–Barre syndrome. Consequently, the prevalence of complaints related to one of the six autonomic COMPASS 31 domains might have been even smaller than in a group of patients, e.g., with diabetic autonomic neuropathy only. Despite this unexpected limitation, the German and the US English COMPASS 31 version yielded closely similar or almost identical scores and sub-scores within the patient group and within the control group (Table 3). Thus, the correlation between the patients’ total scores in the US English and the German Compass 31 was excellent (Pearson’s *r* = 0.934; *p* < 0.001; Fig. 1).

The congruence of scores and sub-scores in the two COMPASS 31 versions is further confirmation that the German version of COMPASS 31 allows for a reliable, valid, and correct evaluation of autonomic complaints in the six COMPASS 31 domains.

Since its publication in 2012, the original US English version of COMPASS 31 has been used in many studies [8–13] and proved to be a reliable and practical self-assessment questionnaire to quantitatively evaluate the autonomic symptoms among patients with various neurological and other disorders [5, 7, 14].

To facilitate the use of the COMPASS 31 questionnaire in non-English-speaking countries, the original version of the COMPASS 31 questionnaire has been translated into several languages, such as Italian [9], Croatian [10], Serbian [10], and Korean [15].

So far, a validated German version of the COMPASS 31 had been missing although it is needed for a more standardized clinical evaluation of patients with autonomic complaints. Validated translations of the questionnaire facilitate the clinical and scientific exchange and cooperation between autonomic research groups in different laboratories and in different countries. The translations provide a required uniform, internationally comparable and still comprehensive questionnaire that delivers valid and reliable data.

Similar to other COMPASS 31 validation studies [9], our study is part of an international project organized by the autonomic group at the Mayo Clinic, Rochester, MN, USA, to translate and validate the original COMPASS 31 version into many different languages (including traditional and simplified Chinese, Japanese, Korean, German, French, Italian, Dutch, Spanish, Portuguese, Swedish, Greek, and others).

As all validated versions of the COMPASS 31, the validated German version may also serve as a clinically useful screening tool that helps determine whether a patient needs specialized autonomic testing with not everywhere available equipment such as continuous blood pressure monitors, hardware, and software for the analysis of heart rate and blood pressure variability, equipment for sudomotor testing, etc. Our control group had a total score of 11.2 ± 9.1 in the German version and of 11.8 ± 8.2 in the US English version. A similarly high score of 10.2 ± 8.9 was reported for the eleven controls of the Italian COMPASS 31 validation study [9]. These relatively high scores might reflect the unspecific nature of autonomic symptoms. The COMPASS 31 questionnaire is well suited to screen for autonomic symptoms, to quantify the individually perceived severity of autonomic symptoms, and to assess longitudinal changes during follow-up studies. Yet, positive findings in the questionnaire necessitate a further diagnostic work-up.

### Limitations of our study

Our study has two limitations. First, as mentioned above, several patients filled out the German or English COMPASS 31 questionnaire before their neurological work-up had been completed, and clinical information, e.g., about urogenital, sweating, or gastrointestinal disorders, was not yet available. Since the study objective focused on assessing the validity of the German answers compared to the English answers, we did not attempt an additional comparison of clinical autonomic findings with the COMPASS 31 scores in the six autonomic sub-domains. Yet, such a comparison might further underline the clinical value of the Compass 31. Second, in the secretomotor domain, we found similar Cronbach’s alpha values for the German (*α* = 0.676) and the English COMPASS 31 version (*α* = 0.536). Yet, these values were somewhat lower than the value reported in the original Mayo Clinic publication (*α* = 0.71) [5]. We assume that the lower secretomotor Cronbach’s alpha values in our German and English version are due to the smaller sample size of our validation study compared to the original Mayo Clinic study that had included 405 participants. Moreover, the secretomotor domain combines questions regarding dry eyes, dry mouth, and abnormal sweating. In the original factor analysis of the COMPASS 31 questionnaire at the Mayo Clinic (Prof. Singer, personal communication, November 11, 2021), the question on sweating was not retained after realizing that it does not necessarily cluster with the “sicca” questions; it is nonetheless included in the final version of COMPASS 31 for reasons of clinical importance. That explains the lower Cronbach alpha of the secretomotor domain.

### Conclusions

In summary, this validation study contributes a German version of the COMPASS 31 that is valid, reliable, internally consistent, and provides results identical to those of the original US English version.

## Supplementary Information

Below is the link to the validated German version of the COMPASS 31.

Supplementary file1 (PDF 31 KB)
