# Supplementary material for: Validation of the Composite Autonomic Symptom Score 31 in the German language
Source: Neurol Sci. 2021 Nov 25;43(1):365–71. doi: 10.1007/s10072-021-05764-4 (PMC8724156; doi:10.1007/s10072-021-05764-4)
Supplement: Supplementary file 1 — Supplementary file1 (PDF 31 KB) [file 10072_2021_5764_MOESM1_ESM.pdf]

Deutsche Version des COMPASS 31 Fragebogens

1. Haben Sie sich im Verlauf des letzten Jahres direkt nach dem Aufstehen aus sitzender oder liegender Position jemals das Gefühl gehabt ohnmächtig zu werden, sich schwindelig oder benommen gefühlt, oder Schwierigkeiten klar zu denken gehabt?
   1 Ja *(falls Sie die Frage mit Ja beantwortet haben, machen Sie bitte mit Frage 2 weiter)*
   2 Nein *(falls Sie die Frage mit Nein beantwortet haben, machen Sie bitte mit Frage 5 weiter)*

2. Wie oft verspüren Sie beim Aufstehen diese Gefühle oder Symptome ?
   1 selten
   2 gelegentlich
   3 häufig
   4 fast immer

3. Wie würden Sie den Schweregrad dieser Gefühle oder Symptome bewerten?
   1 leicht
   2 mäßig
   3 schwer

4. Sind diese Gefühle oder Symptome im Verlauf des letzten Jahres:
   1 Viel schlimmer geworden
   2 Etwas schlimmer geworden
   3 Etwa gleich geblieben
   4 Etwas besser geworden
   5 Viel besser geworden
   6 Vollständig verschwunden

5. Haben Sie innerhalb des letzten Jahres jemals Veränderungen Ihrer Hautfarbe bemerkt, z.B. rot, weiß, oder violett?
   1 Ja *(falls Sie die Frage mit Ja beantwortet haben, gehen Sie bitte zu Frage 6 über)*
   2 Nein *(falls Sie die Frage mit Nein beantwortet haben, gehen Sie bitte zu Frage 8 über)*

6. Welche Körperregionen sind von diesen Farbveränderungen betroffen? *(Bitte alle zutreffenden Antworten ankreuzen)*
   1 Hände
   2 Füße

7. Die Veränderungen Ihrer Hautfarbe:
   1 werden deutlich schlimmer
   2 werden etwas schlimmer
   3 bleiben etwa gleich
   4 werden etwas besser
   5 werden viel besser
   6 sind völlig verschwunden

8. Kam es bei Ihnen <u>innerhalb der letzten 5 Jahre</u> zu Veränderung des Schwitzens Ihres Körpers im Allgemeinen und falls ja, wie würden Sie diese Veränderungen beschreiben?
   1 Ich schwitze viel mehr als früher
   2 Ich schwitze etwas mehr als früher
   3 Ich habe keine Veränderungen im Schwitzen bemerkt
   4 Ich schwitze etwas weniger als früher
   5 Ich schwitze viel weniger als früher

9. Fühlen sich Ihre Augen übermäßig trocken an?
   1 Ja
   2 Nein

10. Fühlt sich Ihr Mund übermäßig trocken an?
   1 Ja
   2 Nein

11. Wenn Sie das Symptombetrachten, das Sie über den längsten Zeitraum beobachtet haben (trockene Augen oder trockener Mund), welche Aussage trifft am besten zu?
   1 Ich hatte keines dieser Symptome
   2 Es wird deutlich schlimmer
   3 Es wird etwas schlimmer
   4 Es bleibt ungefähr gleich
   5 Es wird etwas besser
   6 Es wird viel besser
   7 Es ist vollständig verschwunden

12. Haben Sie <u>im letzten Jahr</u> irgendwelche Veränderungen darin bemerkt, wie schnell Sie beim Essen satt werden?
   1 Ich werde viel schneller satt als früher
   2 Ich werde schneller satt als früher
   3 Ich habe keine Veränderungen bemerkt
   4 Ich werde langsamer satt als früher
   5 Ich werde viel langsamer satt als früher

13. Haben Sie <u>innerhalb des letzten Jahres</u> nach einer Mahlzeit ein übermäßiges Völlegefühl oder anhaltendes Völlegefühl (Gefühl, aufgebläht zu sein) empfunden?
   1 Niemals
   2 Manchmal
   3 Häufig

14. Haben Sie sich <u>innerhalb des letzten Jahres</u> nach einer Mahlzeit übergeben /erbrochen?
   1 Niemals
   2 Manchmal
   3 Häufig

15. Hatten Sie innerhalb des letzten Jahres krampf- oder kolikartige Bauchschmerzen?
  1 Niemals
  2 Manchmal
  3 Häufig

16. Kam es bei Ihnen innerhalb des letzten Jahres zu Durchfällen?
  1 Ja *(falls Sie die Frage mit Ja beantwortet haben, gehen Sie bitte zu Frage 17 über)*
  2 Nein *(falls Sie die Frage mit Nein beantwortet haben, gehen Sie bitte zu Frage 20 über)*

17. Wie oft passiert das?
  1 selten
  2 gelegentlich
  3 häufig, __________ mal pro Monat
  4 ständig

18. Wie schlimm sind diese Durchfälle?
  1 leicht
  2 mäßig
  3 schwer

19. Diese Durchfälle:
  1 werden deutlich schlimmer
  2 werden etwas schlimmer
  3 Bleiben ungefähr gleich
  4 werden etwas besser
  5 werden deutlich besser
  6 sind völlig verschwunden

20. Kam es bei Ihnen innerhalb des letzten Jahres zu Verstopfung?
  1 Ja *(falls Sie die Frage mit Ja beantwortet haben, gehen Sie bitte zu Frage 21 über)*
  2 Nein *(falls Sie die Frage mit Nein beantwortet haben, gehen Sie bitte zu Frage 24 über)*

21. Wie häufig leiden Sie an Verstopfung?
  1 selten
  2 gelegentlich
  3 häufig, __________ mal pro Monat
  4 ständig

22. Wie schwer sind diese Verstopfungen?
  1 leicht
  2 mäßig
  3 schwer

23. Ihre Verstopfung:
    1 wird deutlich schlimmer
    2 wird etwas schlimmer
    3 bleibt ungefähr gleich
    4 wird etwas besser
    5 wird viel besser
    6 ist völlig verschwunden

24. Haben Sie <u>innerhalb des letzten Jahres</u> jemals die Kontrolle über Ihre Harnblasenfunktion verloren?
    1 nie
    2 gelegentlich
    3 häufig, __________ mal pro Monat
    4 ständig

25. Hatten Sie <u>innerhalb des letzten Jahres</u> Probleme, Wasser zu lassen?
    1 nie
    2 gelegentlich
    3 häufig, __________ mal pro Monat
    4 ständig

26. Hatten Sie <u>innerhalb des letzten Jahres</u> Probleme, Ihre Harnblase vollständig zu entleeren?
    1 nie
    2 gelegentlich
    3 häufig, __________ mal pro Monat
    4 ständig

27. Sind Sie <u>innerhalb des letzten Jahres</u> empfindlich gegenüber hellem Licht gewesen, wenn Sie keine Sonnenbrille oder getönte Brille trugen?
    1 Nie *(falls Sie die Frage mit Nie beantwortet haben, gehen Sie bitte zu Frage 29 über)*
    2 gelegentlich
    3 häufig
    4 ständig

28. Wie ausgeprägt ist diese Empfindlichkeit gegenüber hellem Licht?
    1 leicht
    2 mäßig
    3 schwer

29. Hatten Sie innerhalb des letzten Jahres Probleme, mit Ihren Augen etwas scharf zu sehen ?
    1 nie *(falls Sie die Frage mit Nie beantwortet haben, gehen Sie bitte zu Frage 31 über)*
    2 gelegentlich
    3 häufig
    4 ständig

30. Wie ausgeprägt ist dieses Problem, etwas scharf zu sehen?
    1 leicht
    2 mäßig
    3 schwer

31. Das Symptom im Bereich der Augen, das Ihnen die meisten Probleme bereitet (d.h. Empfindlichkeit gegenüber hellem Licht oder scharf zu sehen):

1 Ich habe keines dieser Augen-Probleme
2 Wird deutlich schlimmer
3 Wird etwas schlimmer
4 Bleibt ungefähr gleich
5 Wird etwas besser
6 Wird viel besser
7 Ist vollständig verschwunden
